# The Expression of Inflammasomes NLRP1 and NLRP3, Toll-Like Receptors, and Vitamin D Receptor in Synovial Fibroblasts From Patients With Different Types of Knee Arthritis

**DOI:** 10.3389/fimmu.2021.767512

**Published:** 2022-01-19

**Authors:** Regina Sakalyte, Jaroslav Denkovskij, Eiva Bernotiene, Sigita Stropuviene, Silvija Ona Mikulenaite, Giedrius Kvederas, Narunas Porvaneckas, Vytautas Tutkus, Algirdas Venalis, Irena Butrimiene

**Affiliations:** ^1^ The Clinic of Rheumatology, Traumatology Orthopaedics and Reconstructive Surgery, Institute of Clinical Medicine of the Faculty of Vilnius University, Vilnius, Lithuania; ^2^ State Research Institute Centre for Innovative Medicine, Department of Experimental, Preventative and Clinic Medicine, Vilnius, Lithuania; ^3^ State Research Institute Centre for Innovative Medicine, Department of Regenerative Medicine, Vilnius, Lithuania; ^4^ Department of Chemistry and Bioengineering, The Faculty of Fundamental Sciences, Vilnius Gediminas Technical University, Vilnius Tech, Vilnius, Lithuania; ^5^ Department of Anatomy, Histology and Anthropology, Institute of Biomedical Sciences, Faculty of Medicine, Vilnius University, Vilnius, Lithuania

**Keywords:** arthritis (including rheumatoid arthritis), Toll-like receptor (TLR), vitamin D, metalproteinase, osteoarthristis, inflammasome NLRP, early arthritis (EA), VDR

## Abstract

Activated rheumatoid arthritis (RA) synovial fibroblasts (SFs) are among the most important cells promoting RA pathogenesis. They are considered active contributors to the initiation, progression, and perpetuation of the disease; therefore, early detection of RASF activation could advance contemporary diagnosis and adequate treatment of undifferentiated early inflammatory arthritis (EA). In this study, we investigated the expression of nucleotide-binding, oligomerization domain (NOD)-like receptor family, pyrin domain containing (NLRP)1, NLRP3 inflammasomes, Toll-like receptor (TLR)1, TLR2, TLR4, vitamin D receptor (VDR), and secretion of matrix metalloproteinases (MMPs) in SFs isolated from patients with RA, osteoarthritis (OA), EA, and control individuals (CN) after knee surgical intervention. C-reactive protein, general blood test, anticyclic citrullinated peptide (anti-CCP), rheumatoid factor (RF), and vitamin D (vitD) in patients’ sera were performed. Cells were stimulated or not with 100 ng/ml tumor necrosis factor alpha (TNF-α) or/and 1 nM or/and 0.01 nM vitamin D3 for 72 h. The expression levels of *NLRP1*, *NLRP3*, *TLR1*, *TLR2*, *TLR4*, and *VDR* in all examined SFs were analyzed by quantitative real-time PCR (RT-qPCR). Additionally, the secretion of IL-1β by SFs and MMPs were determined by ELISA and Luminex technology. The expression of *NLRP3* was correlated with the levels of CRP, RF, and anti-CCP, suggesting its implication in SF inflammatory activation. In the TNF-α-stimulated SFs, a significantly lower expression of *NLRP3* and *TLR4* was observed in the RA group, compared with the other tested forms of arthritis. Moreover, upregulation of *NLRP3* expression by TNF-α alone or in combination with vitD3 was observed, further indicating involvement of NLRP3 in the inflammatory responses of SFs. Secretion of IL-1β was not detected in any sample, while TNF-α upregulated the levels of secreted MMP-1, MMP-7, MMP-8, MMP-12, and MMP-13 in all patient groups. Attenuating effects of vitD on the expression of *NLRP3*, *TLR1*, and *TLR4* suggest potential protective effects of vitD on the inflammatory responses in SFs. However, longer studies may be needed to confirm or fully rule out the potential implication of vitD in SF activation in inflammatory arthritis. Both *VDR* and *NLRP3* in the TNF-α-stimulated SFs negatively correlated with the age of patients, suggesting potential age-related changes in the local inflammatory responses.

## 1 Introduction

Onset of inflammatory arthritis is a rheumatic condition that has different outcomes, as reported in different cohort studies: up to 20%–60% of patients will resolve completely even without any treatment, 13%–54% of patients with undifferentiated arthritis (UA) will develop rheumatoid arthritis (RA), 21%–87% UA will persist after 1 year (1). In total, 0.5%–11% UA can be diagnosed with osteoarthritis (OA) (2). Treatment and outcomes of inflammatory rheumatic diseases improved after introduction of “early treatment” strategy and “target” treatment therapy (tumor necrosis factor alpha (TNF-α) inhibitors, interleukin-6 (IL-6) inhibitors, CD20 blockers, etc.) in rheumatology practice, but it still remains unknown, why not all patients receiving this treatment achieve full disease remission (3). Patients with positive rheumatoid factor (RF) and/or anticyclic citrullinated peptide (anti-CCP) and/or C-reactive protein (CRP) are known to be at increased risk of developing worse prognosis of erosive arthritis, the main clinical manifestation of which is synovitis (4). Anti-CCP is known to be one of most sensitive and specific RA diagnostic markers (sensitivity 92.70 [90.67–94.74] and specificity 79.93 [72.44–87.42]) (5). Nevertheless, it can be found positive in other diseases, for instance, approximately 5% of patients with psoriatic arthritis are anti-CCP positive (6, 7). In rare cases, anti-CCP can also be detected as positive in autoimmune hepatitis-1, biliary cirrhosis, hepatitis C virus-related disease, Sjögren’s syndrome (SS), palindromic rheumatism, and systemic lupus erythematosus (SLE) (8, 9). The role of mentioned tests is important but not always enough for early diagnosis and treatment strategy decision-making and needs to be studied further (10). Still, there is no clear link between blood inflammatory and specific immunological laboratory tests and inflammatory processes in synovial fibroblasts (SFs) (11). Therefore, identifying the links between those parameters could lead to a prompt diagnosis of a disease and enable to introduce more successful personalized treatment of various types of early arthritis.

We and others have shown that the SF phenotype differs between forms of arthritis (12, 13). Activated rheumatoid arthritis synovial fibroblasts (RASFs) are among the most important cells promoting RA pathogenesis. They are considered active contributors to the initiation, progression, and perpetuation of the disease (14). In RA, SF transform into aggressive, proliferating RASFs, and along with immune cells, become an invasive, hyperplastic tissue known as pannus, which over time causes cartilage destruction and bone erosion (15). RASFs secrete high levels of proinflammatory cytokines, chemokines, and matrix‐degrading enzymes that stimulate chronic inflammation and lead to progressive, irreversible damage of the diseased joint (16). TNF-α is one of the crucial mediators in the pathogenesis of arthritis, one of the main targets in biological therapy of RA. TNF-α rapidly induces production of matrix metalloproteinases (MMPs) in RASFs that directly contribute to degradation of cartilage components, including collagen type II and proteoglycan aggrecan (17).

Pattern recognition receptors (PRRs), such as Toll-like receptors (TLRs), shown to detect distinct pathogen-associated molecular patterns (PAMPs) or damage-associated molecular patterns (DAMPs), may play a pivotal role in the modulation of joint tissue homeostasis (18) TLRs are predominantly expressed by immune cells; however, their expression has been determined in SFs as well (19, 20). Normally, activation of PPRs is important for adequate inflammatory response; however, if regulatory mechanisms fail, activation of TLRs can lead to uncontrolled local inflammation, trigger a pathological immune response, and lead to inflammatory or autoimmune disorders (21–25). The upregulated expression of TLR2, TLR3, TLR4, TLR5, and TLR7 was found in RASFs compared with those with OA (19) or patients without inflammatory arthritis (26–29). Activation of these receptors in immune cells leads to the upregulation of local inflammatory reactions, including intracellular innate immune sensors nucleotide-binding oligomerization domain (NOD)-like receptors (NLRs), forming multiprotein complexes called inflammasomes, whose activation results in cell pyroptosis and generation of proinflammatory cytokines IL-1α, IL-1β, and IL-18 (30, 31).

NOD-like receptor family pyrin domain containing 1 (NLRP1) was recognized as the first protein from the NLR family to recruit an inflammasome (32), while the NLRP3 inflammasome is currently the most studied one. The oligomerization of NLRP3 inflammasome requires two signals. The first priming signal occurs through membrane receptors, such as TLRs, and *via* activation of the nuclear factor-κB (NF-κB) signaling pathway initiates transcription of *NLRP3* and in turn of *IL-1β* and *IL-18* genes. A second signal is required for activation of inflammasome complex assemblance from NLRP3, adaptor protein apoptosis-associated speck-like protein containing a caspase recruitment domain (ASC) and pro-caspase-1 proteins (31). This may be triggered by diverse stimuli, for instance, extracellular ATP, K^+^ and Ca^2+^ level changes, lysosomal destabilization, mitochondrial dysfunction, reactive oxygen species, uric acid crystals, etc. (31, 33–35). The exact mechanism of NLRP1 inflammasome activation is unknown, but it seems that the NLRP1 protein N-terminal domains must be degraded to promote inflammasome assembly (36). The formation of both mentioned inflammasomes activates caspase-1, which cleaves proinflammatory cytokine progenitor pro-IL-1β and pro-IL-18 into their biologically active forms (31). The activation of the NLRP3 inflammasome contributes to multiple autoimmune diseases, such as ankylosing spondylitis (AS), systemic sclerosis (SSc), SLE, (SS), and RA (31, 33). However, it remains unclear whether their activation might reflect outcomes of early arthritis and potentially become a diagnostic marker facilitating the differentiation of undifferentiated early inflammatory arthritis (EA) into RA, OA or other inflammatory arthritis.

Vitamin D (vitD) is a potential therapeutic molecule, which may prevent or treat inflammatory and possibly degenerative joint diseases (37–39). The active form of vitD binds to specific vitamin D receptors (VDRs), thus activating downstream signaling pathways. Upon vitD binding, VDR dimerizes with the retinoid X receptor to form a heterodimer and afterward attaches to vitD response elements in the promoter regions of various genes (40). In this way, the complex acts as a transcription factor that may regulate more than 900 genes (41). VDR expression has been described in a wide range of different cell types, such as in immune cells, keratinocytes, enterocytes, pancreatic endocrine cells, peripheral blood mononuclear cells (PBMC), etc. (42). Also, VDR expression has been detected in chondrocytes and synoviocytes from inflamed joints of RA subjects (43, 44), suggesting that in addition to the systemic control of the innate and adaptive immune responses, vitD may also be implicated in local joint inflammation (45). Moreover, direct interaction of VDR with NLRP3 at the posttranscriptional level has been demonstrated recently. VDR blocks the association of NLRP3 with BRCC3 deubiquitinase, thus inhibiting the deubiquitination of NLRP3 and afterward suppressing oligomerization and activation of the inflammasome complex (46). Therefore, in the present study, we were seeking to investigate the effect of 1α,25-dihydroxy vitamin D3 (vitD3) on gene expression of innate immune sensors TLRs and NLRPs in SFs of RA, OA, EA, and control individuals (CN).

The main aim of the present study was to investigate the expression of inflammasomes NLRP1 and NLRP3, TLR1, TLR2, TLR4, and VDR in SFs and to compare the data between different patient groups, following stimulation or not with TNF-α and vitD3. We hypothesized that TNF-α stimulates the expression of TLRs and, in turn, inflammasome activation of which further results in proinflammatory activities through the secretion of IL-1β. TNF-α also activates MMPs, which may lead to enhanced catabolic degradation of cartilage and bone. We have also investigated the potential of vitD3 to attenuate the effects of TNF-α and searched for the potential correlations of expression of the tested SF genes with their secretion levels of MMPs, patient age, serum levels of CRP, RF, anti-CCP, and vitD. In parallel, we were interested in investigating whether those processes are differently regulated in various types of arthritis (RA, OA, EA) and CN.

## 2 Materials and Methods

### 2.1 Patients Cohort, Blood Tests, and Synovial Tissue Sample Collection

This study has been approved by the Vilnius Regional Biomedical Research Ethics Committee (Approval No. 158200-18/5-1037-533). Following study protocol, samples were obtained from adult patients (≥18 years) of synovium as remaining tissues after articular replacement surgery in the RA (*n* = 7) and OA (*n* = 4) cases, or during knee arthroscopic synovectomy performed for therapeutic purposes in EA (*n* = 4) cases, or patients who underwent arthroscopy due to meniscus or cruciate ligament traumatic tearing and had no signs of inflammatory arthritis, osteoarthritis, crystal arthropathies, or septic arthritis CN (*n* = 4) cases. All operations were performed by the three senior surgeons. Synovial tissue samples were collected during arthrotomic or arthroscopic knee surgeries under direct visualization. RA was diagnosed based on the American College of Rheumatology (ACR)/European League Against Rheumatism (EULAR) 2010 RA classification criteria (4). OA was diagnosed according to ACR classification criteria for knee OA (47). Patients with inflammatory arthritis at least in one knee, arthritis duration <12 months, and at the moment of surgery, without compliance with the criteria for any inflammatory rheumatic disease were included into the EA group. Patients with other autoimmune diseases, acute inflammation, fever, thyroid disease, diabetes, malignancies, and severe liver and kidney diseases that might have had a huge impact on results were excluded from this study. CRP (elevated if >5 mg/l), general blood test, anti-CCP (positive if >10 U/ml), RF (positive if >30 U/ml), and vitD (normal range 75–100 nmol/l) blood tests were performed to all patients involved in this study. Synovial tissues removed and collected during arthroscopy or surgeries were further processed for cell cultures and analysis.

### 2.2 Synovial Tissue and Cell Culture Preparation

Cells were isolated from synovial tissues as previously described (13). Briefly, mechanically minced synovial tissues were incubated overnight in Dulbecco’s modified Eagle’s medium (DMEM) (with 1 g/L d‐glucose, sodium pyruvate, l‐glutamine, phenol red, Invitrogen) in a humidified 5% CO_2_ incubator at 37°C. After incubation, synovial tissues were digested with 0.1% collagenase (Type I, Biochrom, Cambridge, UK) at 37°C in a shaking mode overnight. Isolated cells were centrifuged at 400×*g* for 10 min and cultured in DMEM supplemented with 10% fetal bovine serum (FBS) (Biochrom), 1% stock solution of penicillin (10,000 units/ml), streptomycin (10 mg/ml), and amphotericin B (0.025 mg/ml, Biological Industries, Haemek, Israel). Passages 2–4 SFs were plated into 25–cm^2^ culture flasks in DMEM containing 10% FBS and stimulated or not for 72 h with or without 100 ng/ml TNF-α (Thermo Fisher Scientific, Waltham, MA, USA) (with additional stimulation after 36 h) and 1 or 0.01 nM of vitD3 (Sigma, St. Louis, MO, USA). At the end of the experimental stimulation, cell culture supernatants were collected under sterile conditions and stored at −80°C until further analysis; cells were lysed in RLT buffer (RNeasy kit, Qiagen, Hilden, Germany) and used for gene expression analysis.

### 2.3 RNA Extraction, cDNA Synthesis, and Quantitative Real-Time PCR

RNA was extracted with RNeasy Mini Spin columns (Qiagen) according to the manufacturer’s instructions, and RNA concentration and purity were measured with the SpectraMax^®^ i3 (Molecular Devices, San Jose, CA, USA) spectrophotometer. Before synthesizing the first complementary DNA strand, RNA samples were treated with DNase I (Thermo Fisher Scientific) and cDNA synthesis was performed with the Maxima^®^First Strand cDNA Synthesis Kit (Thermo Fisher Scientific) according to the manufacturer protocols. qPCRs were performed using the Maxima^®^ Probe qPCR Master Mix (2×) (Thermo Fisher Scientific) and AriaMx real-time PCR system (Agilent Technologies, Santa Clara, CA, USA). The TaqMan^®^ Gene Expression Assays (Applied Biosystems, Waltham, MA, USA) for 8 genes were used for gene expression analysis, using primers as indicated in **Table 1**. The qPCR reaction volume was 25 μl with 0.5 μl of 20× Taqman^®^ Gene Expression Assay mix. All reactions were run in triplicates. Cycle conditions were as follows: initial denaturation step for 10 min at 95°C, followed by 40 cycles of 15 s at 95°C for denaturation and 60 s for annealing and extension. Each RNA sample was controlled for genomic DNA contamination by reactions without reverse transcriptase (RT), and reagent contamination was checked by the reactions without template (NTC). Relative gene expression quantification was calculated using 2^−ΔCT×1,000^ method. The geometric mean of two reference genes—RPS9 and B2M—was used to normalize gene expression. qPCR data were analyzed using AriaMx (Agilent Technologies) software.

**Table 1:** The TaqMan Gene Expression Assays used for gene expression analysis.

Gene Assay ID	Encoded Protein
*RPS9* Hs02339424_m1	40S ribosomal protein S9
*B2M* Hs00984230_m1	Beta-2 microglobulin
*TLR-1* Hs00413978_m1	Toll-like receptor 1
*TLR-2* Hs02621280_s1	Toll-like receptor 2
*TLR-4* Hs00152939_m1	Toll-like receptor 4
*VDR* Hs01045843_m1	Vitamin D receptor
*NLRP1* Hs00248187_m1	PYD domain-containing protein 1
*NLRP3*Hs00918082_m1	PYD domain-containing protein 3

### 2.4 Detection of Secreted Proteins by Luminex and ELISA Assays

Analysis of IL-1β concentration in nondiluted supernatants was performed using commercially available ELISA kit (R&D Systems, Minneapolis, MN, USA); levels of MMP-1, MMP-7, MMP-8, MMP-12, and MMP-13 were measured using Luminex Technology and ProcartaPlex Human MMP-Panel 5 plex panel (Affymetrix, eBioscience, San Diego, CA, USA), according to manufacturer’s instruction. Cell culture medium was used for background normalization.

### 2.5 Statistical Analysis

The descriptive statistics (mean values ± standard deviation, median, and lower and upper ranges) were applied for data analysis. The variables were tested for normality using the Kolmogorov–Smirnov test. After evaluating the sample size, nonparametric statistics small sample size has been applied. For the comparisons between the groups, medians were compared using the Mann-Whitney *U* test. The related samples were compared using a nonparametric Wilcoxon signed-rank test. Correlations between all cohort data were calculated using Spearman’s nonparametric correlation test. *p*-values less than 0.05 were considered statistically significant. Data were analyzed using Graphpad Prism v. 9 and SPSS version 20 (IBM Corp, Armonk, NY, USA) software.

## 3 Results

### 3.1 Patient Enrollment and Characterization

A total of 19 patients (7 in RA, 4 in OA, 4 in EA, 4 in CN group) were included in the study after informed consent was signed. Mean age was 53.1 years ( ± 11.9), and 14 (73.7%) were females. No differences in age or sex were detected between different pathology patient groups. In total, 10 (52.6%) patients were RF positive and 9 (47%) were anti-CCP positive. All RA patients were anti-CCP and RF positive, EA patients were either RF or/and anti-CCP positive, and all OA and CN patients were negative for both anti-CCP and RF. RA group had the highest value of CRP (*p* < 0.05). Normal vitD level in 4 (21.05%) patients’ sera was found (**Table 2**). In all study cohort (*n* = 19) patients, RF showed correlation with anti-CCP (*r* −0.869, *p* < 0.000), CRP (*r* −0.628, *p* −0.004), as well as anti-CCP and CRP (*r* −0.539, *p* < 0.017). No correlation was found between age and serum levels of RF, anti-CCP, CRP, and vitD.

**Table 2:** Baseline demographic, laboratory tests, and treatment history characteristics.

Characteristic	All Patients	EA	RA	OA	CN
	*N* = 19	*N* = 4	*N* = 7	*N* = 4	*N* = 4
Females (*N* (%)]	14 (73.7%)	2 (50%)	6 (85.7%)	4(100%)	2 (50%)
Age (years: mean (SD)]	53.1 (11.9)	40.5 (7.4)	62.29 (7.3)	60.75 (2.87)	42.0 (5.1)
Anti-CCP [positive *N* (%)]	9 (47%)	2 (50%)	7 (100%)	0	0
Anti-CCP [(CU), median]	12.6	70.7^*^	1,610.5^*^	–	–
Anti-CCP (min-max)	4.1–2,776.8	4.6–199.6	12.6–2,776.8	–	–
RF [positive *N* (%)]	10 (52.6%)	3 (75%)	7 (100%)	0	0
RF [(IU/ml) median]	23.2	102.8	110.1	–	–
RF [(IU/ml) min-max]	20.0–1,221.5	20.0– 597.6	23.2–1,221.5	–	–
CRB [(mg/l), mean (SD)]	7.1 (7.5)	3.5 (3.9)	13.9 (7.9)	2.2 (3.1)	3.6 (3.3)
CRB [(mg/l) median]	4.8	2.6^**^	17.1^**^	0.9^**^	2.97^**^
CRB [(mg/l), min-max]	0.1–22.3	0.63–9.12	1.5–22.3	0.1–6.7	0.98–7.8
VitD (nmol/l)					
Normal result (%)	4 (21.05%)	1 (25.0%)	2 (28.6%)	1 (25%)	0 (0%)
Mean (SD)	51.14 (29.2)	54.4 (30.8)	49.99 (37.1)	61.01 (29.6)	39.97 (15.2)
Ever DMARD treatment [*N* (%)]	7 (36.8)	1 (25)	7 (100)	0	0
Ever used TNF-α [*N* (%)]	3 (15.8)	0	3 (42.9)	0	0
Ever used RTX [*N* (%)]	2 (10.5)	0	2 (28.6)	0	0
Ever used MTX [*N* (%)]	4 (21.1)	1 (25)	3 (42.9)	0	0
GK	7 (36.8)	0	7 (100)	0	0

EA, early arthritis; RA, rheumatoid arthritis; OA, osteoarthritis; CN, control group; SD, standard deviation; Anti-CCP, anticyclic citrullinated peptide; CU, chemiluminescent units; CRP, C-reactive protein; RF, rheumatoid factor; DMARD, disease-modifying antirheumatic drugs; TNF-α, tumor necrosis factor alpha; RTX, rituximab; MTX, methotrexat; GK, glucocorticosteroid. ^*^p < 0.05 (RA compared with EA group); ^**^p < 0.05 (RA compared with EA, OA, and CN groups). CRP (elevated if >5 mg/l), general blood test, anti-CCP (positive if >10 U/ml), RF (positive if >30 U/ml), and vitD (normal range 75–100 nmol/l).

All patients in the RA group had a history of disease-modifying antirheumatic drug (DMARD) treatment; one patient in the EA group was treated with methotrexate [following early arthritis treatment EULAR strategy (48)] (**Table 2**).

### 3.2 Synovial Fibroblast Analysis

**Figure 1:**
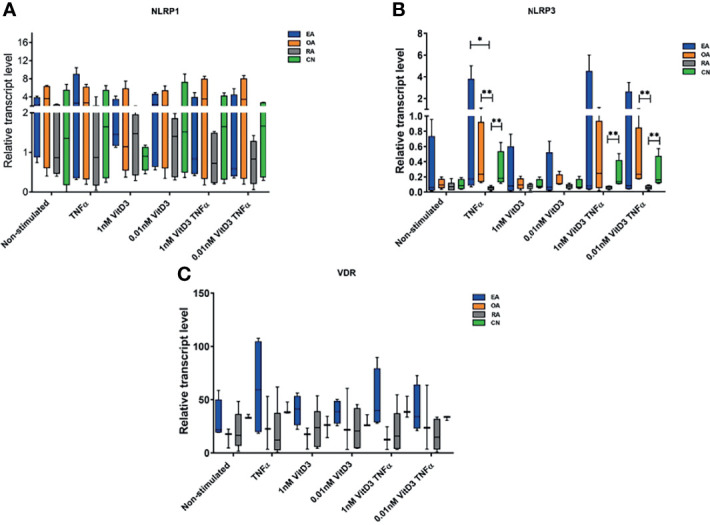
Relative NOD-like receptor family, pyrin domain containing (NLRP)1 **(A)** and NLRP3 **(B)** inflammasome and vitamin D receptor (VDR) **(C)** gene expression levels. Synovial fibroblasts from patients with undifferentiated early inflammatory arthritis (EA), osteoarthritis (OA), rheumatoid arthritis (RA), and control individuals (CN) were cultured for 72 h with or without stimulation with 100 ng/ml tumor necrosis factor α (TNF-α), 1 or 0.01 nM 1α,25-dihydroxy vitamin D3 (vitD3), or their combination. The relative gene expression quantification was calculated using 2^−ΔCT×1,000^ methodology. Geometric means of two control genes, 40S ribosomal protein S9 (RPS9) and beta-2 microglobulin (B2M) were used to normalize gene expression. The box length represents the interquartile range with a median. The whiskers represent the minimum and maximum data values. ^*^ *p* < 0.05; ^**^ *p* < 0.01.

**Figure 2:**
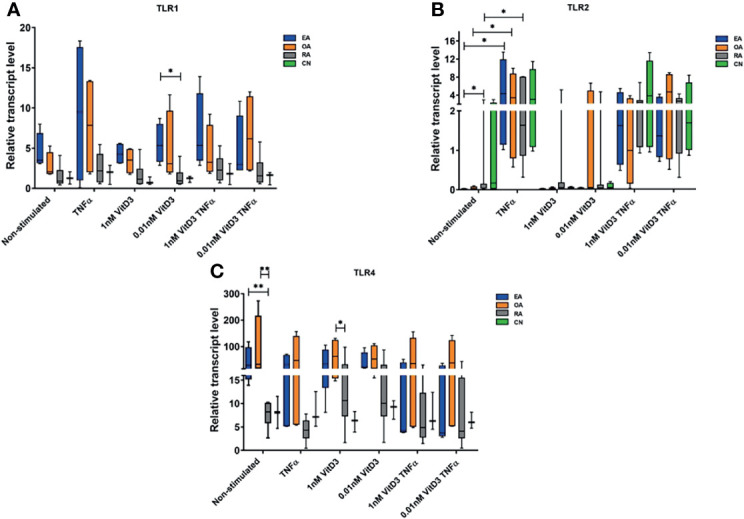
Relative gene expression levels of Toll-like receptor (TLR)1 **(A)**, TLR2 **(B)**, and TLR4 **(C)**. Synovial fibroblasts from patients with undifferentiated early inflammatory arthritis (EA), osteoarthritis (OA), rheumatoid arthritis (RA), and control individuals (CN) were cultured for 72 h with or without stimulation with 100 ng/ml tumor necrosis factor α (TNF-α), 1 or 0.01 nM 1α,25-dihydroxy vitamin D3 (vitD3), or their combination. The relative gene expression was calculated using 2^−ΔCT×1,000^ method. Geometric means of two control genes, 40S ribosomal protein S9 (RPS9), and beta-2 microglobulin (B2M) were used to normalize gene expression. The box length represents the interquartile range with a median. The whiskers represent the minimum and maximum data values. ^*^ *p* < 0.05; ^**^ *p* < 0.01.

**Figure 3:**
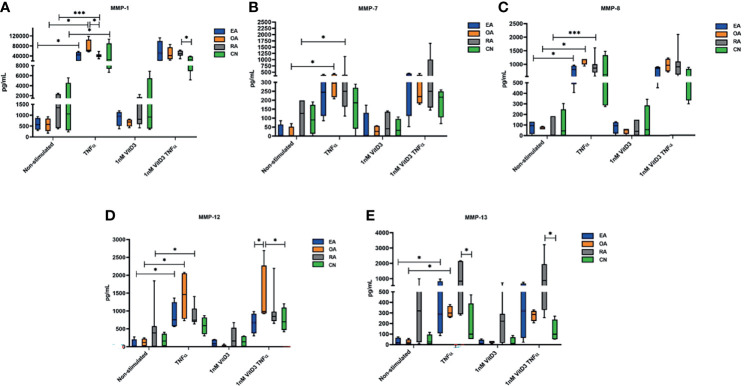
Secretion of matrix metalloproteinases (MMP)-1 **(A)**, MMP-7 **(B)**, MMP-8 **(C)**, MMP-12 **(D)**, and MMP-13 **(E)**, determined by Luminex technology in culture supernatants of synovial fibroblasts from patients with undifferentiated early inflammatory arthritis (EA), osteoarthritis (OA), rheumatoid arthritis (RA), and control individuals (CN) after 72 h stimulation with 100 ng/ml tumor necrosis factor α (TNF-α) or 1 nM 1α,25-dihydroxy vitamin D3 (vitD3) or in supernatants of cell culture cultivated in Dulbecco’s modified Eagle’s medium (DMEM) supplemented with 1% antibiotics and 10% fetal bovine serum (control). The box length represents the interquartile range with a median. The whiskers represent the minimum and maximum data values. ^*^ *p* < 0.05; ^***^ *p* < 0.001.

The expression of *VDR*, *TLR1*, *TLR2*, *TLR4*, *NLRP1*, and *NLRP3* inflammasome genes and secretion of MMP-1, MMP-7, MMP-8, MMP-12, and MMP-13 in SF cultures and after stimulation at different vitD3 doses (0.01 or 1 nM) or TNF-α 100 ng/ml alone or their combination was measured in all patients. Data were analyzed between the related nonstimulated and stimulated samples in the whole study cohort (**Tables 3**–**5**) and compared between the different pathology patient groups (**Figures 1**–**3**).

**Table 3:** Effects of the stimulation with TNF-α and VitD on the expression of NLRP1 and NLRP3 inflammasomes and *VDR* genes in synovial fibroblast cultures of study individuals (*N* = 16–18).

Analyzed Gene	Stimulation	Relative Transcript Level [median (range)]
NLRP1	Nonstimulated	1.23 [0.01–6.76]
	TNF-α 100 ng/ml	1.04 [0.05–10.43]
	1 nM vitD3	1.17 [0.29–7.47]
	0.01 nM vitD3	1.55 [0.31–9.03]
	1 nM vitD3 TNF-α 100 ng/ml	0.87 [0.18–8.57]
	0.01 nM vitD3 TNF-α 100 ng/ml	0.7 [0.06–8.7]
NLRP3	Nonstimulated	0.08 [0.01–0.96]^*^
	TNF-α 100 ng/ml	0.13 [0.01–5.01]^*^
	1 nM vitD3	0.08 [0.01–0.76]
	0.01 nM vitD3	0.09 [0.01–0.66]
	1 nM vitD3 TNF-α 100 ng/ml	0.09 [0.01–5.95]
	0.01 nM vitD3 TNF-α 100 ng/ml	0.11 [0.02–3.42]^*^
VDR	Nonstimulated	21.86 [1.77–58.76]
	TNF-α 100 ng/ml	26.17[0.74–107.66]
	1 nM vitD3	24.32 [3.61–56.33]
	0.01 nM vitD3	25.9 [3.46–60.66]
	1 nM vitD3 TNF-α 100 ng/ml	29.4 [3.3–89.67]
	0.01 nM vitD3 TNF-α 100 ng/ml	29.47 [0.82–72.63]

NLRP, NOD-like receptor family, pyrin domain containing; VDR, vitamin D receptor; TNF-α, tumor necrosis factor alpha; vitD3, 1α,25-dihydroxy vitamin D3. Nonparametric Wilcoxon signed-rank test: ^*^p-value <0.05 (compared with nonstimulated and stimulated-related samples).

**Table 4:** Effects of the stimulation with TNF-α and VitD on the expression of *TLR1*, *TLR2*, and *TLR4* genes in synovial fibroblast cultures of study individuals (*N* = 17–19).

Analyzed Gene	Stimulation	Relative Transcript Level [median (range)]
TLR1	Nonstimulated	1.87 [0.41–7.97]^*^
	TNF-α 100 ng/ml	2.85 [0.08–18.32]^*,^‡
	1 nM vitD3	1.73 [0.49–5.60]
	0.01 nM vitD3	1.79 [0.5–11.65]
	1 nM vitD3 TNF-α 100 ng/ml	2.86 [0.46–13.91]^*^
	0.01 nM vitD3 TNF-α 100 ng/ml	2.23 [0.43–11.99]^*.‡^
TLR2	Nonstimulated	0.03 [0.003–3.00]^*^
	TNF-α 100 ng/ml	1.64 [0.32–13.54]^*^
	1 nM vitD3	0.03 [0.01–4.99]
	0.01 nM vitD3	0.01 [0.01–6.49]
	1 nM vitD3 TNF-α 100 ng/ml	1.47 [0.02–13.30]^*^
	0.01 nM vitD3 TNF-α 100 ng/ml	1.55 [0.3–8.81]^*^
TLR4	Nonstimulated	11.52 [2.67–272.82]^*^
	TNF-α 100 ng/ml	5.84 [0.43–156.4]^*,‡^
	1 nM vitD3	11.7 [1.68–130.88]
	0.01 nM vitD3	15.45 [1.69–110.73]
	1 nM vitD3 TNF-α 100 ng/ml	5.46 [1.5–155.41]^*^
	0.01 nM vitD3 TNF-α 100 ng/ml	5.12 [0.47–142.23]^*,‡^

TLR, Toll-like receptor; TNF-α, tumor necrosis factor alpha; vitD3, 1α,25-dihydroxy vitamin D3. Nonparametric Wilcoxon signed-rank test: ^*^p-value <0.05 (compare nonstimulated and stimulated related samples); ^‡^p-value <0.05 (compare stimulated TNF-α 100 ng/ml and stimulated TNF-α 100 ng/ml with 0.001 nMvitD3-related samples).

**Table 5:** The levels of MMP-1, MMP-7, MMP-8, MMP-12, and MMP-13 secretion in supernatants of synovial fibroblast cultures of the studied cohort (*N* = 19).

MMP	Stimulation	Median [Range] (pg/ml)
MMP-1	Nonstimulated	586.18 [177.32–5,567.35]^***^
	TNF-α 100 ng/ml	43632.13 [6,666.88–118,020.06]^***^
	1 nM vitD3	815.26 [347.74–6876.54]
	1 nM vitD3 TNF-α 100 ng/ml	41103.14 [5,184.93–112,105.89]^***^
MMP-7	Nonstimulated	0.00 [0.00–198.92]^***^
	TNF-α 100 ng/ml	245.55 [0.00–1,126.93]^***^
	1 nM vitD3	0.00 [0.00–172.45]
	1 nM vitD3 TNF-α 100 ng/ml	241.4 [52.87–1,649.88]^***^
MMP-8	Nonstimulated	58.68 [0.00–303.18]^***^
	TNF-α 100 ng/ml	906.86 [275.33–1,605.19]^***^
	1 nM vitD3	58.68 [0.00–343.53]
	1 nM vitD3 TNF-α 100 ng/ml	821.58 [296.3–2,082.79]^***^
MMP-12	Nonstimulated	162.73 [0.00–1,842.93]^**^
	TNF-α 100 ng/ml	743.13 [302.63–2,078.4]^**^
	1 nM vitD3	0.00 [0.00–675.1]
	1 nM vitD3 TNF-α 100 ng/ml	847.49 [302.63–2,689.64]^**^
MMP-13	Nonstimulated	33.08 [0.00–993.27]^***^
	TNF-α 100 ng/ml	328.7 [54.31–2,173.26]^***^
	1 nM vitD3	28.1 [0.00–722.72]
	1 nM vitD3 TNF-α 100 ng/ml	292.51 [22.64–3,217.84]^***^

MMP, matrix metalloproteinase; TNF-α, tumor necrosis factor alpha; vitD3, 1α,25-dihydroxy vitamin D3. Nonparametric Wilcoxon signed-rank test: ^*^p-value < 0.05; ^**^p-values < 0.01; ^***^p < 0.001 (comparison of MMP secretion levels (Luminex technology) in 72 h nonstimulated and stimulated related samples).

#### 3.2.1 Expression of *NLRP1* and *NLRP3* Inflammasomes and *VDR* Gene in Synovial Fibroblasts

Expression of *NLRP1*, *NLRP3*, and *VDR* genes was detected in SFs of all patients enrolled into the study (**Table 3**); the levels were similar in all tested pathology groups without stimulation (**Figure 1**). Stimulation with TNF-α and/or 0.01 or 1 nM of vitD3 had no effect on the expression of *NLRP1* and *VDR* in neither whole cohort nor in either group separately. However, under stimulation with TNF-α or TNF-α and 0.01 nM vitD3, the expression of the *NLRP3* was higher in the whole study cohort. Under stimulation with TNF-α alone, expression of the *NLRP3* gene tended to increase in all pathology groups, except RA. Consequently, stimulation with TNF-α resulted in a significantly higher expression of *NLRP3* in CN, OA, and EA patient groups, as compared with the RA group. In the presence of vitD3, stimulation with TNF-α resulted in similar although somewhat less-expressed differences between the patient groups. *VDR* was similarly expressed in all the groups of this study.

#### 3.2.2 Expression of *TLR1*, *TLR2*, and *TLR4* Genes in Synovial Fibroblasts

Expression of *TLR1*, *TLR2*, and *TLR4* genes was detected in SFs of all patients enrolled into the study. Stimulation with TNF-α alone or in combination with vitD3 (at doses 0.01 or 1 nM) resulted in increase of *TLR1* and *TLR2* and decrease of *TLR4* expression related samples in all study cohort (*p* < 0.05). Stimulation with 0.01 or 1 nM vitD3 alone did not affect *TLR1*, *TLR2*, and *TLR4* gene expression, but related sample analysis revealed that 0.01 nM vitD3 attenuated effects of TNF-α on *TLR1* and *TLR4* gene expression (*p* < 0.05) (**Table 4**).

Further expression of *TLR1*, *TLR2*, and *TLR4* genes was analyzed in SFs of different pathology patient groups. Without stimulation, the expression of *TLR2* gene was significantly higher in the RA, as compared with the EA patients group, while the expression of *TLR4* was 3.9- and 3.5-fold lower in the RA, as compared with the EA and OA groups, respectively. The differences between OA and RA groups were also observed in the presence of 1 nM vitD3 stimulation. Somewhat similar results were obtained for *TLR1* gene expression; however, the differences between the groups did not reach the level of statistical significance, except between the EA and RA groups under stimulation with vitD3. Seventy-two-hour cell stimulation with TNF-α resulted in significant upregulation of *TLR2* gene expression in the EA, OA, and RA groups. Similar effects were observed in both, at the absence or the presence of vitD3 stimulation (**Figure 2**).

#### 3.2.3 Secretion of MMPs and IL-1β by Synovial Fibroblasts

Secretion of MMP-1, MMP-7, MMP-8, MMP-12, and MMP-13 was analyzed in SFs of all 19 patients enrolled into the study in related sample analysis. After stimulation with TNF-α, secretion of all MMPs was statistically significantly increased. A similar effect was observed after stimulation with TNF-α and 1 nM vitD3. Difference between stimulation with TNF-α alone or TNF-α in combination with 1 nM vitD3 MMP secretion was not significant in the related sample analysis. Stimulation with 1 nM vitD3 alone had no effect on MMP level (**Table 5**).

Secretion of MMPs without and under stimulation with TNF-α, 1 nM vitD3, and TNF-α with 1 nM vitD3 was further analyzed in different pathology patient group SFs (**Figure 3**). No differences were confirmed in patient groups in all MMP secretions in SFs without stimulation. Stimulation with 1 nM vitD3 alone had no influence on expression of all tested MMPs in different pathology SFs. Following stimulation with TNF-α, the levels of MMP-1 were significantly increased in all tested groups (EA, OA, RA, CN). MMP-1 secretion was higher in SFs of the OA group, as compared with the RA (*p* < 0.05). Levels of MMP-7 were upregulated in the OA (*p* < 0.05) and RA (*p* < 0.05) groups, and levels of MMP-8 and MMP-12 were upregulated in the EA, OA, and RA groups (*p* < 0.05). Under stimulation with TNF-α, the secretion of MMP-13 increased in all groups, although the increase was statistically significant only in the EA and OA groups. Furthermore, under stimulation with TNF-α, the levels of MMP-13 were statistically higher in the RA, as compared with the CN group. Stimulation with 1 nM vitD3 alone had no effect on levels of MMPs in SFs of different pathology patient groups. Combined stimulation with 1 nM vitD3 and TNF-α had relatively similar effects on the expression of MMPs to those of stimulation with TNF-α alone.

Secretion of IL-1β, the cytokine regulated through activation of inflammasome, was also analyzed. No traces of IL-1β were detected in supernatants of either tested group even after stimulation with TNF-α, although the ELISA test was chosen with sensitivity as low as 1 pg/ml.

### 3.3 Analysis of Correlation Between Characteristics of Synovial Fibroblasts and Patient Age, Serum Levels of CRP, RF, anti-CCP, and vitD

Correlations between *NLRP1*, *NLRP3*, *TLR1*, *TLR3*, *TLR4*, and *VDR* gene expression levels, MMP-1, MMP-7, MMP-8, and MMP-12 secretion levels in nonstimulated and stimulated with TNF-α SF samples, patient age, serum levels of CRP, RF, anti-CCP, and vitD were analyzed in the whole study cohort. Confirmed significant correlations are presented in **Table 6**. Correlations between serum levels of CRP, anti-CCP, RF, and vitD are described in Section 3.1. No correlations were detected between VitD and tested gene expression in SF and MMP secretion levels.

**Table 6:** Analysis of correlation between characteristics of synovial fibroblasts and patient age, serum levels of CRP, RF, anti-CCP, and vitD in whole patient cohort^a^.

	MMP-12 TNF-α	MMP-13 NS	MMP-13 TNF-α	Other MMPs^***^	*VDR* TNF-α	*VDR* NS	*TLR4* TNF-α	*TLR4* NS	*TLR2* TNF-α	*TLR2* NS	*TLR1* TNF-α	*TLR1* NS	*NLRP3* TNF-α	*NLRP3* NS	*NLRP1* TNF-α	*NLRP1* NS
**Age**					−0.594^*^								−0.516^*^			
**Anti-CCP**								−0.583^*^					−0.746^**^	−0.498^*^		
**RF**														0.518^*^		
**VitD**																
**CRP**		0.472^*^	0.644^*^										−0.606^**^			
***NLRP1* NS**					0.593^*^		0.579^*^	0.615^*^	0.647^**^		0.594^*^				0.911^**^	
***NLRP1* TNF-α**					0.689^**^		0.703^**^	0.571^*^	0.723^**^		0.618^*^					
***NLRP3* NS**													0.482^*^			
***NLRP3* TNF-α**					0.561^*^		0.703^**^	0.609^*^								
***TLR1* NS**								0.752^**^								
***TLR1* TNF-α**					0.807^**^		0.600^*^	0.733^**^	0.662^**^							
***TLR2* NS**								0.525^*^								
***TLR2* TNF-α**					0.774^**^		0.532^*^									
***TLR4* NS**	0.487^*^				0.632^*^	0.647^**^	0.637^**^									
***TLR4* TNF-α**					0.739^**^											
***VDR* NS**					0.644^**^											
***VDR* TNF-α**																

a Only statistically significant results are presented after whole study cohort (19 patients) data correlation analysis.

Anti-CCP, anti-cyclic citrullinated peptides; RF, rheumatoid factor; VitD, vitamin D; CRP, C-reactive protein; NS, nonstimulated; TNF-α, after stimulation with 100 ng/ml TNF-α; NLRP, NOD-like receptor family pyrin domain containing; TLR, Toll-like receptor; VDR, vitamin D receptor; MMP, metalloproteinases. ^*^p ≤ 0.05; ^**^p ≤ 0.01; ^***^Other MMP (MMP-1, MMP-7, MMP-8) did not correlate with TLRs and NLRPs.

In statistical analysis, patient age had negative correlations with *VDR* and *NLRP3* gene expression levels in TNF-α-stimulated samples (**Table 6**).

*NLRP1* gene expression levels have any correlation with serum laboratory tests. *NLRP1* gene expression in the nonstimulated and stimulated with TNF-α SF samples correlate with the expression of *TLR1*, *TLR2*, *TLR4*, and *VDR* in TNF-α in stimulated and *TLR4* nonstimulated with TNF-α SF samples (**Table 6**).

*NLRP3* gene expression levels correlate with serum anti-CCP and RF level in nonstimulated samples, whereas TNF-α-stimulated SF samples correlate with serum anti-CCP and CRP level. *NLRP3* gene expression levels in nonstimulated samples do not present statistically significant correlation with other analyzed gene expression levels. After stimulation with TNF-α, *NLRP3* gene expression levels correlate with gene expression levels in *TLR4* in nonstimulated and stimulated with TNF-α and *VDR* in stimulated with TNF-α SF samples (**Table 6**).

*VDR* gene expression levels have any correlation with serum laboratory tests. *VDR* and *TLR4* gene expression levels correlate in nonstimulated SF samples. *VDR* gene expression levels in stimulated with TNF-α SF samples correlate with the *TLR1*, *TLR2*, *TLR 4*, *NLRP1*, and *NLRP3* gene expression levels (**Table 6**).

In the correlation analysis between *TLR* gene expression levels and serum laboratory tests, only *TLR4* gene expression in nonstimulated samples correlates with serum anti-CCP levels. In the correlation analysis between *TLR* gene expression and MMP secretion levels, only one correlation was confirmed between *TLR4* gene expression in nonstimulated and MMP-12 stimulated with TNF-α samples. Correlation analysis between *TLRs*, *NLRPs*, and *VDR* in nonstimulated and stimulated with TNF-α SF samples gene expression level is described above in this section. There were also multiple correlations between expressions of different *TLRs* (**Table 6**).

No significant correlations between MMP secretion levels and *NLRPs* and *VDR* gene expression levels were confirmed. Only MMP-13 stimulated with TNF-α had a weak correlation with *TLR4* in nonstimulated SFs. In the correlation analysis between MMP secretion levels and serum laboratory tests, only MMP-13 secretion in nonstimulated SF samples correlated with serum CRP levels (**Table 6**).

## 4 Discussion

Long-term acute inflammation-related phenotypic changes in RASFs lead to pannus formation and increased cartilage and bone tissue degradation (12, 49). Activated RASFs promote inflammation of the synovial tissue and thus maintain the autoimmune process by expressing adhesion molecules, secreting inflammatory cytokines and matrix-degrading enzymes (50). Therefore, development of the inflammatory joint disease was shown to depend not only on external factors but also on changes in functional properties of SF (51). Pathological reactions might arise from the inappropriate response of the receptor, for instance, TLR or NLRP, due to their particular genetic background or to the inappropriate quantity or quality of ligands (52).

Inflammasomes are functionally involved in inflammatory responses of multiple cell types, including fibroblasts from different origins, i.e., gingival, lung, liver, heart, skin, and cancer-associated fibroblasts (53–55). In the present study, we aimed to investigate if inflammasomes were associated with the inflammatory responses of SFs during different forms of arthritis. TNF-α is one of the essential mediators of inflammation in RA (56, 57), so we ought to better understand how it contributes to the aggressive phenotype of RASFs. In the present study, the cells were stimulated with 100 ng TNF-α as suggested in previous publications (58, 59). VDR plays an important role in limiting the inflammatory phenotype in a mouse model of RA (60) and was shown to block the activation of inflammasomes (46). Therefore, we hypothesized that vitD signaling pathway can attenuate inflammatory activation of RASFs through modulation of inflammasome activation pathway. The present study determined the expression of both *NLRP1* and *NLRP3* in SFs of all patients involved, suggesting their potential role in the synovial tissue.

Although very little data exist, NLRP1 inflammasome is likely to be implicated in the pathogenesis of RA. For instance, inhibition of P2X4 receptor and 11β-hydroxysteroid dehydrogenase 1 was shown to attenuate activation of inflammasome NLRP1 and expression of IL-1β in both collagen-induced arthritis (CIA) model and synovial cells of patients with RA (61, 62). However, we found no statistically significant difference in *NLRP1* gene expression between the tested groups in the present study. Stimulation with neither TNF-α nor vitD3 had any effect on the *NLRP1* inflammasome expression in SFs of all the analyzed groups, which suggests that those factors do not directly regulate NLRP1 activation. However, gene correlation analysis of all patients involved in this study showed that the *NLRP1* in both nonstimulated or TNF-α-stimulated cells is positively correlated with *TLR1*, *TLR2*, *TLR4*, and *VDR* gene expression in TNF-α-stimulated cells. These data suggest that these genes are implicated in synovial responses together though.

NLRP3 reacts to a diverse set of PAMPs and DAMPs, which may contribute to the etiopathogenesis of inflammatory disease through inflammasome activation (63). Increased expression and function of NLRP3 inflammasome were previously reported in the peripheral blood cells of RA patients (63). TNF-α acts as a priming signal, which induces expression of NLRP3 mRNA and IL-1β mRNA in RASFs (64). The expression of synovial NLRP3 was positively correlated with clinical and radiographic arthritis scores in the CIA model in mice (65); however, the synovial tissue, which was analyzed in that study, contains several cell types. In the present study, we were focused on the role of NLRP3 exclusively in SFs, which are considered the aggressive drivers of local inflammation in the joint. In our study, the expression of *NLRP3* was correlated with the levels of CRP, RF, and anti-CCP, suggesting its implication in SF inflammatory activation. Moreover, significant upregulation of *NLRP3* expression by TNF-α alone or TNF-α in combination with 0.01 nM vitD3 was observed, further indicating involvement of NLRP3 in the inflammatory responses of SFs. Although the combination of TNF-α with 1 nM of vitD3 resulted in somewhat upregulation of *NLRP3* expression, the change was not significant, suggesting that higher dose of vitD3 might have played a protective role.

The lower *NLRP3* expression after TNF-α stimulation in the RA patient group suggests an imbalance of this system activation in the acute inflammatory state. However, it is complicated to evaluate whether this dysregulation is a consequence of a long-term inflammation, and medication prescribed to RA patients or rather patients with this imbalance are more prone to develop RA. In other cells from patients with RA, monocyte, neutrophil, and inflammasome activation results in IL-1β and IL-18 secretion (65, 66). Therefore, we were seeking to determine if altered NLRP3 activation in TNF-α-stimulated SFs of the RA group results in functional responses. Although there are some data on IL-1β secretion by SF (67), we found no traces of its presence in supernatants of SFs. These data suggest that the inflammasome complex may not have assembled in our cells, even though *NLRP3* gene expression was observed. This is in agreement with the previously reported data, where in fibroblast-like synoviocytes from patients with RA function, the inflammasome complex was not formed even though NLRP3 mRNA was detected (68). The explanation could be that the signal received through membrane receptor activation is sufficient to upregulate the expression of the *NLRP3* gene, but protein expression and additional cellular or molecular stimulation is required for further inflammasome complex assembly and afterward inflammasome-activated proinflammatory cytokine secretion (31). Various molecules, such as crystals, and fragments of apoptotic cells, which might act as potential contributors to NLRP3 inflammasome activation *via* the second signal, are detected in the OA- or RA-affected joint (34, 35). However, the lack of these molecules in our *in vitro* experimental setting could have led to the absence of IL-1β secretion by SFs; therefore, further research should be conducted to investigate the effects of complex NLRP3 formation in SFs. Noteworthy, even after the formation of the inflammasome complex, IL-1β secretion may be impaired due to inefficient *IL-1β* gene expression and translation or caspase-1 activation.

It has been demonstrated that interaction between vitD and TLRs could have a mutual form, and activation of TLRs may affect the expression of the *VDR* gene in direct and indirect manners. For instance, stimulation of human monocytes by appropriate TLR1/2 ligands leads to increased expression of VDR and CYP27B1 (VD-activating enzyme 1α-hydroxylase) (69). Also, TLR1/2 ligands increased the expression and functions of VDR in human monocytes (70) and increased antimicrobial functions of human macrophages in a vitD-dependent manner (71). Our study revealed correlations of *VDR* expression with TLRs and inflammasomes *NLRP1* and *NLRP3*, particularly under stimulation with TNF-α. The moderate positive correlation between *VDR* and *NLRP3* after stimulation with TNF-α indicates that VDR may inhibit NLRP3 inflammasome assembly eventually at the protein level. Furthermore, both *VDR* and *NLRP3* in the TNF-α-stimulated SFs negatively correlated with the age of patients, suggesting potential age-related changes in inflammatory responses.

Our results demonstrate that *TLR1*, *TLR2*, and *TLR4* gene expression are characteristic for SFs and were detected in all analyzed patient groups. Furthermore, we found higher levels of *TLR2* in RA as compared with EA, which only partially corresponds to the results of previous studies, where significantly higher TLR2 mRNA levels were demonstrated in both early and long-standing RA (72–75). In the present study, TNF-α significantly upregulated *TLR2* gene expression in the EA, OA, and RA groups but not in the CN group. However, *TLR2* gene expression levels in unstimulated RA and OA samples were similar. Also, only the expression of the *TLR2* gene was significantly higher in the RA than in the EA patients group but not the expression of *TLR1* or *TLR4*. Although TNF-α stimulated *TLR1* expression, differences between the stimulated and nonstimulated samples reached a significance level only when *TLR1* gene expression was compared in the related samples of the whole cohort. Such differences seem to be associated with a small number of patients in the groups divided by diagnoses. Higher expression levels of *TLR4* were found in the OA and EA groups compared with the RA group, which is controversial to the data of recent studies where *TLR4* expression was statistically significantly higher in the RA group than OA (75, 76). On the other hand, not all studies uniformly confirm the association of TLR4 with RA pathogenesis. For instance, similar expression levels of *TLR1* and *TLR4* genes in RA and OA were reported (77). Also, no differences in the baseline expression levels of *TLR2*, *TLR4*, and *TLR9* were observed (73). In addition, *TLR4* gene expression was upregulated by any of the stimuli used. Moreover, a slight, but reproducible decrease in *TLR4* gene expression was observed after stimulation with IL-1β, TNF-α, and synthetic bacterial lipopeptide (sBLP) (73). Similar results were found in the present study, where TNF-α significantly reduced *TLR4* expression (**Table 4**). Reasons for these discrepancies might be differences in the patient cohorts examined and might be related to clinically active disease duration or to variations in the level of inflammation *in vivo* due to prolonged RA treatment. Noteworthy, upregulated expression of TLR4 is not only specific for RA. TLR4 is implicated in many other inflammatory diseases such as psoriasis, inflammatory bowel disease, and SLE. Also, all TLRs, including TLR1 and TLR4, are present and upregulated in OA cartilage and synovium, particularly in the lower limbs such as knee and hip cartilage (25, 73, 77, 78). In the present study, the expression of the *TLR4* gene in the OA group was significantly higher than in the RA group. Similar results were determined for *TLR1* expression. Previous studies have shown that in the OA synovial fluid, TLR4 is also present in soluble form, and it is associated with the OA severity as well (79, 80). The fact that TLR4 and TLR1 may be involved in the pathogenesis not only of RA but also of other forms of arthritis, including OA, is also indicated by the correlation analysis between clinical parameters and TLR gene expression. In the present study, a statistically significant negative correlation was determined between anti-CCP and *TLR4* expression levels, potentially due to a separation of patients with early or long arthritis duration.

It has been shown that vitD has a dual effect on the expression and functions of TLRs in immune and nonimmune cells; however, the effect of vitD has been mainly studied in cells of the immune system. For instance, several investigations approved the roles of vitD in the downregulation of *TLR2* and *TLR4* in human pathologic proinflammatory conditions. For instance, vitD decreased expression of *TLR2* and *TLR4* on the monocytes of the patients suffering from type 2 diabetes mellitus and latent autoimmune diabetes (81). It has also been reported that calcitriol decreased *TLR2* and *TLR4* expression and downstream proinflammatory cytokines in human keratinocytes (82). Similar results were observed examining the effect of vitD on *TLR2* (83, 84) and *TLR4* (83, 85, 86) expression. On the other hand, the opposite effect of vitD has also been shown. For instance, increased TLR2/1-dependent expression of cathelicidin against *Mycobacterium tuberculosis* was demonstrated (87). Increased expression and functions of TLR2 (88), TLR3 (89), and TLR8 (90) by vitD to protect injuries from infections have also been documented. Despite the abundance of studies, we were unable to find previous data on the effect of vitD on SFs. In our study, vitD-attenuated TNF-α modulated expression of *TLR1* and *TLR4* and show similar modulation tendency for *NLRP3*. It may likely be related to a short period of stimulation. SFs with vitD3 were stimulated for only 3 days. Also, the preventive effect of the vitD could be more substantial if cells at first would be stimulated with vitD3 and then with TNF-α.

We were also seeking to identify associations of altered *NLRP3* expression with other functional activities of SFs. The activation of TLRs initiates the production of destructive tissue enzymes, as metalloproteinases, the main factors involved in the degradation of bone and cartilage during RA. Previous studies have demonstrated that MMP-1 secretion was higher in the RA (including early RA) groups compared with the OA or non-RA (other inflammatory arthritis like psoriatic arthritis (PsA) and AS) (91). Another study showed no differences in MMP-1 secretion between the OA patients and healthy controls (92). In the present study, the stimulation with TNF-α resulted in higher levels of MMP-1 in the OA, as compared with the RA group. MMP-13 is the most prominent MMP in RA (93). We also found that MMP-13 secretion was highest in the RA group as compared with the other groups.

No effect of vitD on MMP-1 secretion in SFs of the RA patients has been previously reported (94). On the other hand, in rat articular cartilage model, low vitD diet resulted in elevated MMP-13 levels, while vitD supplementation significantly decreased MMP-13 levels (95). In the OA cartilage, the effect of vitD3 alone on MMP-1 production was hardly evident, but when TNF-α was added, more marked responses were observed (96). We have also demonstrated that stimulation with TNF-α has increased the secretion of all MMPs in all groups, whereas vitD3 had no significant effects on their levels.

Taken together, we determined the expression of both *NLRP1* and *NLRP3* in all tested samples of SFs. Stimulation with TNF-α showed no effects on the *NLRP1*, whereas significantly upregulated the expression of *NLRP3*. When comparing different forms of arthritis, significantly lower expression of *NLRP3* under stimulation with TNF-α was observed in the RA group compared with all other tested groups, suggesting potential contribution of altered NLRP3 activation to the etiopathogenesis of advanced RA. The differences in expression of *NLRP3* and *TLR4* in EA and RA highlight the importance of long-lasting inflammation or potentially long-lasting treatment on the activation of this pathway. Our data do not confirm the functional outcomes of NLRP3 activation in SFs, i.e., IL-1β secretion or association with levels of MMPs, although we cannot rule out that the drawbacks of the *in vitro* system, i.e., lack of secondary signal may have led to the inefficient formation of inflammasome complex and in turn lack of functional signaling. Attenuating effects of vitD on the expression of *NLRP3*, *TLR1*, and *TLR4* in SFs suggest potential protective effects of vitD on the inflammatory responses. In contrast, it may be worth to investigate longer or preventive stimulation, prior to the application of TNF-α, which could have revealed even more evident effects of vitD. Noteworthy, both *VDR* and *NLRP3* in the TNF-α-stimulated SFs negatively correlated with the age of patients, suggesting potential age-related changes in inflammatory responses.

In conclusion, we raise the hypothesis that better understanding of inflammasome activation in synovial fibroblasts and possible effects of vitamin D could help to modulate outcomes of early and advanced arthritis.

## Data Availability Statement

The raw data supporting the conclusions of this article will be made available by the authors, without undue reservation.

## Ethics Statement

The studies involving human participants were reviewed and approved by Vilnius Regional Bioethics Committee (Approval No. 158200-18/5-1037-533, date of approval 2018-05-08). The patients/participants provided their written informed consent to participate in this study.

## Author Contributions

RS, SS, GK, NP, and VT: patient selection and enrolment into the study, clinical data and blood sample collection, and data analysis and interpretation. EB, SS, and IB: study conception. RS, JD, and SM: cell culture experiments, sample data analysis, and interpretation. RS, JD, SM, EB, and SS manuscript preparation. All authors: manuscript editing. AV and IB: critical revision. All authors contributed to the article and approved the submitted version.

## Funding

This study was funded by the Research Council of Lithuania; grant ID number P-MIP-17-192.

## Conflict of Interest

The authors declare that the research was conducted in the absence of any commercial or financial relationships that could be construed as a potential conflict of interest.

## Publisher’s Note

All claims expressed in this article are solely those of the authors and do not necessarily represent those of their affiliated organizations, or those of the publisher, the editors and the reviewers. Any product that may be evaluated in this article, or claim that may be made by its manufacturer, is not guaranteed or endorsed by the publisher.

## Abbreviations

Anti-CCP, anticyclic citrullinated peptide; AS, ankylosing spondylitis; ASC, apoptosis-associated speck-like protein containing a caspase recruitment domain; B2M, beta-2 microglobulin; CIA, collagen-induced arthritis; CN, control; CRP, C-reactive protein; CU, chemiluminescent unit; DAMP, damage-associated molecular pattern; DMARD, disease-modifying antirheumatic drug; EA, undifferentiated early inflammatory arthritis; ELISA, enzyme-linked immunosorbent assay; GK, glucocorticosteroid; IL, interleukin; MMP, matrix metalloproteinase; MTX, methotrexate; NLR, NOD-like receptor; NLRP, NOD-like receptor family pyrin domain containing; NOD, nucleotide-binding oligomerization domain; OA, osteoarthritis; PAMP, pathogen-associated molecular pattern; PBMC, peripheral blood mononuclear cell; PRR, pattern recognition receptor; PsA, psoriatic arthritis; RA, rheumatoid arthritis; RASF, rheumatoid arthritis synovial fibroblast; RF, rheumatoid factor; RPS9, ribosomal protein S9; RTX, rituximab; SF, synovial fibroblast; SLE, systemic lupus erythematosus; SS, Sjögren’s syndrome; SSc, systemic sclerosis; TLR, Toll-like receptor; TNF-α, tumor necrosis factor alpha; VDR, vitamin D receptor; VitD, vitamin D; VitD3, 1α,25-dihydroxy vitamin D3; UA, undifferentiated arthritis.
