# Supplementary material for: The Expression of Inflammasomes NLRP1 and NLRP3, Toll-Like Receptors, and Vitamin D Receptor in Synovial Fibroblasts From Patients With Different Types of Knee Arthritis
Source: Front Immunol. 2022 Jan 19;12:767512. doi: 10.3389/fimmu.2021.767512 (PMC8807559; doi:10.3389/fimmu.2021.767512)
Supplement: Supplementary file 1 [file DataSheet_1.pdf]

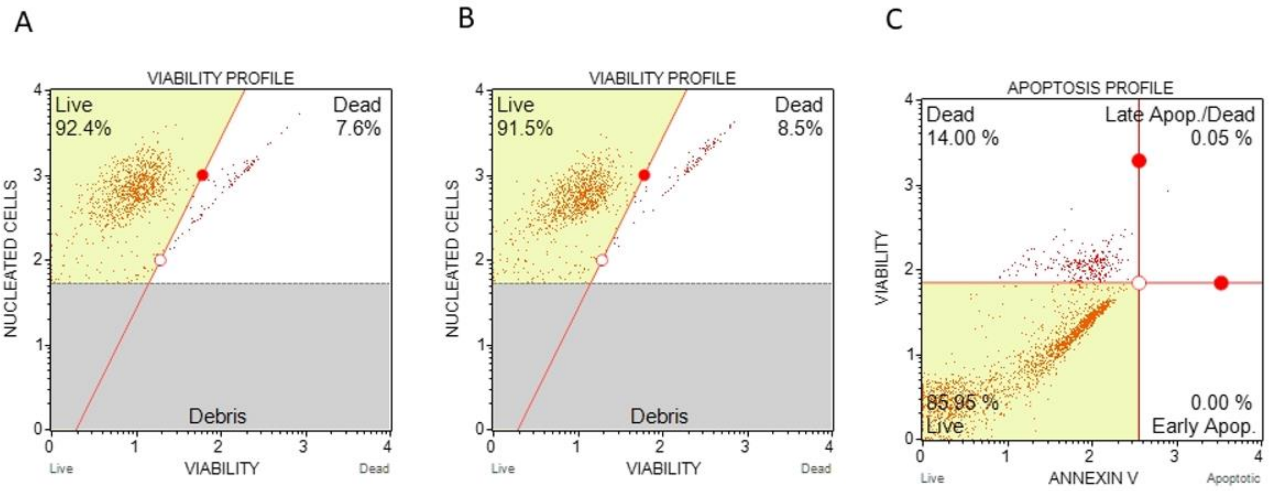

Fig. Z. Synovial fibroblasts cells viability after stimulation with 100 ng/ml TNF α.

The number of viable cells, type of cell, cell death, and stage of apoptosis were analyzed using the Muse ® Annexin V & dead cell (Merck Millipore) and Muse ® Count & Viability assays (Merck Millipore) following the manufacturer's instructions. The Muse™ Cell Analyzer (Merck Millipore) was used to quantitative identification of live, early and late apoptotic, and dead cells by measuring cell fluorescence intensity. Figure X. Measurement of synovial fibroblasts viability and apoptosis. Representative viability profiles of control (A) and 100ng/ml TNF α (B) stimulated cell population. C Representative fibroblasts apoptosis profile of 100ng/ml TNF α stimulated cell population. Identification of live, early and late apoptotic and dead cells was investigated by Muse™ Cell Analyzer after 3 days of stimulation. Non-stimulated cells were grown in parallel for the same time period (Figure Z).
